# Layered precision suturing vs. traditional double-layer closure at cesarean: a randomized trial of uterine scar healing and maternal outcomes

**DOI:** 10.3389/fsurg.2026.1749613

**Published:** 2026-05-04

**Authors:** Minjie Zhao, Xiaoyu Yang, Peng An, Wenjing Hu, Xiaojuan Wang, Huimin Xing, Heding Zhang

**Affiliations:** Department of Hand Numbnes, Shijiazhuang Maternal and Child Health Hospital, Shijiazhuang, Hebei Province, China

**Keywords:** cesarean delivery, residual myometrial thickness, scar healing, uterine closure, uterine niche

## Abstract

**Background:**

Optimal uterine closure at cesarean delivery remains unsettled, as most comparative trials emphasize the number of layers rather than surgical biomechanics. We tested whether a layered precision (LP) suturing technique with quantified tension control improves uterine scar healing compared with traditional double-layer (DL) closure.

**Methods:**

We conducted a single-center, parallel-group, superiority randomized trial (1:1 LP vs. DL) at a tertiary obstetric hospital. Eligible participants were women aged 20–40 years with a singleton pregnancy at ≥37 weeks of gestation who were scheduled for a first cesarean delivery with a planned low-transverse uterine incision and who provided written informed consent. Participants were randomized using concealed allocation, and cointerventions were standardized. Surgeons were aware of the assigned closure technique, whereas imaging outcome assessors and data analysts remained blinded to the assigned interventions. A total of 500 participants were recruited and randomized. The primary endpoint at 6 months was poor healing on standardized ultrasound, defined as residual myometrial thickness (RMT) < 2.2 mm or a uterine niche (indentation ≥2 mm). Secondary endpoints included continuous imaging metrics [RMT, 3D scar morphology, MRI diffusion assessed by the apparent diffusion coefficient (ADC), and optional shear-wave elastography], perioperative outcomes, patient-reported outcomes (PROs), and subsequent pregnancy events. Analyses were conducted on an intention-to-treat basis using prespecified adjusted models.

**Results:**

Of the 500 participants recruited and randomized, primary-outcome data were available for 425 participants (LP 212; DL 213). Poor healing occurred in 16 of 212 participants (7.5%) in the LP group vs. 32 of 213 participants (15.0%) in the DL group [absolute risk difference=7.5 percentage points, 95% CI 1.5–13.4; risk ratio (RR) 0.50, 95% CI 0.28–0.89; *P* = 0.015; adjusted RR 0.50, 95% CI 0.28–0.89]. Mean RMT was greater in the LP group (3.05 ± 0.62 vs. 2.78 ± 0.65 mm; adjusted difference 0.27 mm, 95% CI 0.12–0.42; *P* < 0.001) and niche prevalence was lower (12/212 vs. 24/213; RR 0.50, 95% CI 0.26–0.98; *P* = 0.043). In subset analyses, LP showed higher MRI ADC values (1.18 ± 0.12 vs. 1.12 ± 0.13 × 10⁻^3^ mm^2^/s; difference 0.06, 95% CI 0.02–0.10; *P* = 0.004) and lower elastography stiffness [28 [24–33] vs. 32 [27–37] kPa; difference −4, 95% CI −6 to −2; *P* = 0.001]. Perioperative outcomes modestly favored LP (median estimated blood loss 480 vs. 520 mL, *P* = 0.009; operative time 42.5 vs. 45.3 min, *P* < 0.001), with similar rates of fever, infection, and adverse events [any adverse event (AE) 42/250 vs. 49/250; serious AE 4/250 vs. 6/250].

**Conclusion:**

LP closure with quantified tension improved 6-month uterine scar healing across multimodal imaging without introducing new safety concerns, supporting a shift from “stitch count” to a standardized, biomechanics-informed technique.

## Introduction

Cesarean delivery is one of the most commonly performed operations worldwide, and its prevalence continues to rise across regions (1, 2). These trends amplify the importance of technical choices during uterine closure, which may influence both immediate perioperative recovery and long-term uterine integrity (1, 2). Despite the ubiquity of cesarean birth, uterotomy closure remains heterogeneous. Contemporary surveys and guideline evidence reviews show wide variation in practice and a lack of consensus regarding the optimal technique (3, 4). Even with recent randomized data, including the 3-year follow-up of the 2Close trial, traditional double-layer (DL) closure has not demonstrated superiority over single-layer closure for key reproductive outcomes, underscoring that commonly used methods may not fully address scar quality (5). A recent *AJOG* review by Bujold and Romero further emphasized that uterine closure outcomes are influenced not only by stitch configuration but also by tissue biology, intraoperative hemostasis without ischemia, restoration of uterine anatomy, and the biomechanical handling of the hysterotomy. That review synthesized the biological rationale and clinical implications of uterine closure techniques while highlighting the need for prospective clinical studies to directly evaluate standardized, biomechanics-informed operative approaches (6).

Downstream morbidity increasingly centers on the uterine niche (cesarean scar defect), a myometrial indentation at the scar site that may present with abnormal uterine bleeding, pelvic pain, or subfertility, and in subsequent pregnancies may be associated with cesarean-scar pregnancy, placenta accreta spectrum, and uterine rupture or dehiscence (7, 8). Recent consensus work formalized cesarean scar disorder as the presence of a niche with specific symptoms, while a niche is generally defined sonographically as an indentation ≥2 mm, which facilitates cross-study comparability but does not, by itself, guide prevention or optimal repair (7, 8).

Accurate, multidimensional assessment of healing is therefore essential. Transvaginal and three-dimensional ultrasound are first-line modalities for characterizing residual myometrial thickness (RMT), niche presence, and scar morphology, with large contemporary cohorts and method papers supporting their utility (9). Complementary modalities can provide additional tissue characterization when needed: pelvic MRI, including diffusion-weighted imaging sequences and apparent diffusion coefficient (ADC) mapping, helps evaluate scar morphology and surrounding tissues, while emerging elastography may provide quantitative stiffness metrics that reflect remodeling biology (10–12). Collectively, these tools enable reproducible, quantitative assessment of scar healing, which may inform both surgical technique evaluation and perioperative management strategies (9–12).

Yet, a methodological gap persists at the operating table. Most comparative studies have emphasized stitch pattern (single- vs. double-layer) without standardizing *layer-specific tissue handling* or quantifying suture tension—factors that directly influence tissue perfusion, apposition, and healing quality. Current surveys and evidence reviews explicitly highlight the lack of consensus and the opportunity for technique standardization grounded in surgical biomechanics (3–5, 13, 14). Accordingly, the present study was designed not to replicate the conceptual framework of the recent review but to extend it through a prospective, surgical-focused, randomized evaluation of a standardized operative protocol. Specifically, our trial evaluates whether a layered precision (LP) suturing approach, tailored to the deep and superficial myometrium and implemented with quantified intraoperative tension control, translates biological and biomechanical principles into measurable improvements in uterine scar healing with direct relevance to operative practice. The primary objective was to determine whether this approach reduces poor uterine wound healing at 6 months compared with traditional double-layer closure. Consistent with contemporary definitions, poor healing will be assessed primarily by ultrasound, defined as decreased RMT and/or the presence of a uterine niche (indentation ≥2 mm). Secondary endpoints include perioperative outcomes, advanced imaging metrics (MRI with diffusion parameters and optional elastography), patient-reported outcomes, and subsequent pregnancy events (7–12).

## Methods

### Study design and oversight

We conducted a prospective, two-arm, parallel-group, superiority randomized controlled trial (1:1) comparing layered precision suturing with traditional double-layer uterine closure at cesarean delivery. Participants were enrolled preoperatively after screening for eligibility and provision of written informed consent. Allocation was concealed using a computer-generated permuted block randomization sequence with a fixed block size of 4, stratified by site, and implemented via sequentialally numbered, opaque envelopes or a secure web-based randomization system opened after hysterotomy and immediately before uterine closure. The trial design included explicit measures to minimize bias: surgeons were not blinded due to the nature of the intervention, while participants, imaging outcome assessors, and data analysts remained blinded until completion of the primary analyses. The protocol included prospective trial registration, institutional review board approval, and written informed consent. This design enabled evaluation of both surgical technique and perioperative outcomes.

### Setting and training

The trial was conducted at Hebei Provincial Maternal and Child Health Hospital (Shijiazhuang, Hebei, China), with ethical approval granted by the Institutional Review Board of the same institution (IRP HPH202544091U). Preparatory activities [protocol finalization, IRB approval, standard operating procedure (SOP) development, staff training, and EDC build] were conducted from June to December 2022; formal training/certification of surgeons and imaging/laboratory staff was completed before the first patient enrollment. Patient enrollment and study surgeries took place from January to December 2023; the 6-month primary imaging follow-up was completed by December 2024, and long-term follow-up and final analyses continued through December 2025. Surgeons completed a standardized workshop that included checklists and video-based SOPs for both techniques, while imaging and laboratory personnel were certified in protocol procedures before enrollment to ensure uniform acquisition and measurement standards. A total of eight attending obstetric surgeons participated in the trial, all of whom had prior experience performing cesarean deliveries and completed study-specific training before trial initiation. Surgeons performed procedures in both randomized groups according to the allocation sequence. The median number of study procedures per surgeon was 62 (range 54–71), reflecting balanced participation across operators. This approach minimizes interoperator variability and helps ensure that observed differences are attributable to technique rather than surgeon experience. The study adhered to the principles of research ethics, with written informed consent obtained from all participants; results were disseminated through peer-reviewed publications and scientific meetings, and deidentified data and analysis code were shared in accordance with institutional policy following publication.

### Participants

Inclusion criteria were women aged 20–40 years with a singleton pregnancy at ≥37 weeks’ gestation who were scheduled for a first cesarean delivery with a planned low-transverse uterine incision, regardless of whether the cesarean was elective or emergent. A “planned low-transverse uterine incision” was defined as a preoperative or intraoperative decision to perform a standard low-transverse incision as the intended uterine entry approach. Women requiring alternative uterine incisions (e.g., classical vertical, T-shaped, or fundal) were excluded. Exclusion criteria included multiple gestation, uterine malformation, emergent conversion for fetal or maternal indications, active infection, coagulopathy or long-term anticoagulation, major intraoperative complications requiring expanded repair, and contraindications to MRI. In addition, maternal baseline characteristics potentially relevant to wound healing were prospectively recorded at enrollment, including diabetes mellitus, body mass index (BMI), smoking status, hypertensive disorders, anemia, and other clinically relevant comorbidities. These data support both baseline comparability and adjustment for confounders affecting perioperative or scar outcomes. These variables were summarized by study group at baseline to assess comparability after randomization and were incorporated into adjusted analyses to evaluate their potential impact on healing outcomes.

### Interventions

In the LP arm, the deep myometrium was approximated with interrupted stitches using a target needle travel of approximately 1.5 cm, and the superficial myometrium was closed with a continuous stitch using approximately 1.0 cm travel with serosal inversion. Intraoperative suture tension was quantified and capped at ≤30 N using a handheld digital tension gauge to minimize ischemic compression and optimize tissue apposition. The 30 N threshold was selected based on preliminary feasibility testing using uterine tissue models and simulated suturing, which indicated that higher tensions produced excessive tissue compression, while lower tensions provided stable approximation without over-compression. The device was calibrated according to the manufacturer's instructions before the trial, and surgeons were trained to ensure reproducible tension measurements. While feasible in the trial setting, the generalizability of this tension cap to routine practice and other devices remains to be validated. In the traditional DL arm, surgeons performed a standard full-thickness uterine closure followed by a continuous serosal layer according to the institutional SOP. Prior to suturing, the wound area was prepared and disinfected under sterile conditions. Tissue approximation was performed using 4-0 nylon mounted on a 26-mm, 3/8-circle reverse-cutting needle. Sutures were placed using an interrupted technique to ensure appropriate tissue approximation while minimizing tension at the wound edges. The spacing and depth of each stitch were adjusted according to tissue thickness to achieve stable closure and optimal wound healing. All procedures were performed by an experienced clinician who followed the same SOP throughout the study to ensure procedural consistency across participants. All participating surgeons were trained to perform both closure techniques before study initiation and adhered to the same operative framework, differing only in the randomized closure method. This approach helps ensure that observed differences are attributable to the closure technique rather than other intraoperative factors. Across both arms, we recorded suture type, needle size, closure time, intraoperative events, and estimated blood loss (EBL) (suction volume and the gauze-weight method). Perioperative cointerventions, including antibiotic prophylaxis, uterotonics, thromboprophylaxis, multimodal analgesia, wound care, and early mobilization, were standardized to reduce confounding. These measures enhance the trial's relevance to perioperative surgical outcomes.

### Outcomes

The primary endpoint at 6 months postpartum was poor uterine wound healing, defined as RMT <2.2 mm or the presence of a uterine niche on transvaginal/3D ultrasound, consistent with contemporary niche terminology (indentation ≥2 mm) and reporting practices (7, 15).

Key secondary outcomes included perioperative events assessed from the intraoperative period until hospital discharge and, for postoperative infectious morbidity, through the first 30 postoperative days. These perioperative events included estimated blood loss and operative time recorded intraoperatively, postoperative fever ≥38°C assessed during hospitalization, and wound infection defined according to CDC criteria assessed during hospitalization and at postoperative follow-up within 30 days; ultrasound-based imaging outcomes, including scar morphology and 3D scar volume (3–5, 9); patient-reported outcomes (pain, menstrual changes, and sexual quality of life at discharge, 6 weeks, and 6 months); subsequent pregnancy outcomes (interpregnancy interval, placenta accreta spectrum, uterine rupture/dehiscence, mode of birth, and neonatal outcomes); and exploratory mechanistic markers [histology and immunohistochemistry at the incision edge, inflammatory markers such as IL-6 and C-reactive protein (CRP), and targeted microbiome profiling]. Pelvic MRI and shear-wave elastography were prespecified as exploratory adjunct imaging outcomes rather than key secondary outcomes.

### Imaging protocol and measurement

At 6 months, all participants underwent transvaginal and 3D ultrasound with standardized acquisition. RMT was measured at the thinnest segment of the scar, and the presence of a niche was defined as a sonographic indentation threshold of ≥2 mm, with 3D reconstructions for volumetric morphology where applicable (9, 15). Pelvic MRI and shear-wave elastography were prespecified as optional exploratory adjunct imaging modalities and were not required for assessment of the primary endpoint. Pelvic MRI was performed in a subset of participants who completed the 6-month follow-up, had no contraindications to MRI, and were able to undergo imaging within the predefined study window. Shear-wave elastography was performed in participants attending the same follow-up visit when equipment and a trained operator were available, and image quality was adequate for analysis. Because these modalities were optional, feasibility-dependent, and conducted in limited subsets, MRI- and elastography-based analyses were considered exploratory and hypothesis-generating rather than definitive between-group comparisons. Pelvic MRI (T2-weighted and diffusion-weighted sequences) was obtained in a subset to characterize tissue signal and calculate the ADC as a quantitative marker complementing ultrasound (10). Shear-wave elastography was optional and, when available, and was used to quantify scar stiffness as an emerging correlate of remodeling biology (12).

### Patient-reported outcomes and follow-up

Clinical assessments occurred at 48–72 h, 6 weeks, and 6 months. Validated PRO instruments [with predefined scoring and minimal clinically important differences (MCIDs)] were administered at discharge, 6 weeks, and 6 months. To reduce reporting bias in these subjective outcomes, participants remained blinded to group allocation during the primary 6-month follow-up period whenever feasible, and PRO data were collected using standardized questionnaires by study staff who were not involved in the surgical procedure and were unaware of treatment assignment. Long-term surveillance for up to 5 years was conducted through scheduled outpatient follow-up, structured telephone contact, and review of hospital medical records, and this approach was used to capture subsequent pregnancy occurrence, interpregnancy interval, obstetric complications, mode of delivery, uterine rupture or dehiscence, placenta accreta spectrum, and neonatal outcomes, when available. Standardized case-report forms and prespecified follow-up windows were used to promote consistency in long-term outcome ascertainment across participants. Participant petention was supported through reminders, travel reimbursement, and flexible imaging windows.

### Sample size

The target sample size was *N* = 500 (≈250/arm). The sample size calculation was based on an assumed poor-healing rate of 15% in the control group, derived from published literature on cesarean scar healing and uterine niche prevalence assessed by follow-up ultrasound using comparable outcome definitions and follow-up intervals (8, 14). Because the layered precision technique was designed to improve myometrial apposition and reduce excessive suture-related tissue compression, an absolute reduction to 7.5% was considered a clinically meaningful and plausible treatment effect for the primary endpoint. This anticipated effect size was based on the hypothesized benefit of a standardized biomechanics-informed closure technique rather than on a formal pilot study. Assumptions include a control poor-healing rate of 15%, a target of 7.5% (absolute reduction 7.5%), a two-sided *α* of 0.05 (), and 80% power; an assumed attrition rate of approximately 15% was incorporated to account for loss to follow-up, incomplete 6-month imaging assessments, and other missing primary outcome data. Sensitivity analyses using multiple imputation and per-protocol (PP) datasets were prespecified to assess the robustness of results and potential bias introduced by missing data. Accordingly, the minimum per-arm size was inflated to 250 to preserve power under the anticipated loss to follow-up.

### Randomization, masking, and bias mitigation

Participants were randomized 1:1 by concealed allocation, as described. Surgeons could not be blinded due to the nature of the uterine closure procedure; however, participants, imaging outcome assessors, and analysts handling PROs and imaging endpoints remained blinded until completion of primary analyses. Participants were not informed of the specific uterine closure technique used, and study personnel responsible for collecting patient-reported outcomes were not involved in the surgical procedure and were unaware of treatment allocation during the primary follow-up period. Imaging acquisition followed standardized SOPs. Two independent radiologists, blinded to group allocation, graded ultrasound/MRI outcomes, with adjudication of discordance and assessment of inter-rater reliability (intraclass correlation). Analysts handling PROs and imaging endpoints were also blinded to group assignment.

### Data capture, quality assurance, and monitoring

Data were recorded in a secure electronic data-capture system with range/logic checks, periodic source data verification, and monitored query resolution. Imaging quality assurance included periodic scanner-parameter checks and reproducibility audits; laboratory assays used duplicates and control curves.

### Statistical analysis plan

The primary analysis followed the intention-to-treat principle and compared the proportion of participants with poor healing using risk ratios with 95% confidence intervals (*χ*^2^ or log-binomial/Poisson with robust variance). Baseline characteristics were summarized by randomized group to assess comparability, including clinical factors potentially affecting wound healing, such as diabetes mellitus, obesity, smoking status, hypertensive disorders, anemia, and other maternal comorbidities. Continuous variables were presented as mean ± standard deviation, and categorical variables were presented as frequencies and percentages. Standardized mean differences (SMDs) were calculated to evaluate balance between groups, with values <0.10 considered indicative of adequate baseline comparability. Prespecified covariates (age, BMI, parity, indication, and incision length) were included in adjusted models. In addition, diabetes mellitus, obesity, smoking status, and other clinically relevant maternal comorbidities were incorporated into adjusted analyses and in the interpretation of healing outcomes to assess their potential impact on uterine scar healing. Continuous endpoints (RMT, ADC, elastography) were analyzed using linear models, categorical endpoints were analyzed using risk or odds ratios, and time-to-event methods were applied for long-term obstetric outcomes. Because multiple surgeons performed procedures during the trial, exploratory sensitivity analyses evaluated the consistency of treatment effects across surgeons. Surgeon identifier was examined as a clustering factor in secondary models to assess potential operator-level variability and learning-curve effects. Analyses of MRI- and elastography-based outcomes were prespecified as exploratory, as these optional imaging modalities were performed only in feasibility-based subgroups, and the trial was not specifically powered to detect between-group differences for these endpoints. Missing data were addressed using multiple imputation, and per-protocol sensitivity analyses were performed. Multiplicity across key secondary outcomes was controlled using methods such as the Holm procedure. All statistical analyses were performed using JMP version 16.1 (SAS Institute, Cary, NC, USA). A two-sided *p* value <0.05 was considered statistically significant, unless otherwise specified for multiplicity-adjusted secondary analyses. In light of contemporary randomized evidence questioning whether conventional closure configuration alone determines long-term reproductive outcomes, a standardized, biomechanics-informed uterine closure technique warrants evaluation beyond stitch number alone (5, 16).

### Safety monitoring

Adverse events (AEs) and serious AEs (hemorrhage, infection, readmission, reoperation) were defined *a priori*. A Data and Safety Monitoring Board reviewed accumulating data on a scheduled basis with prespecified stopping boundaries for harm and futility.

## Results

### Participant flow

**Figure 1:**
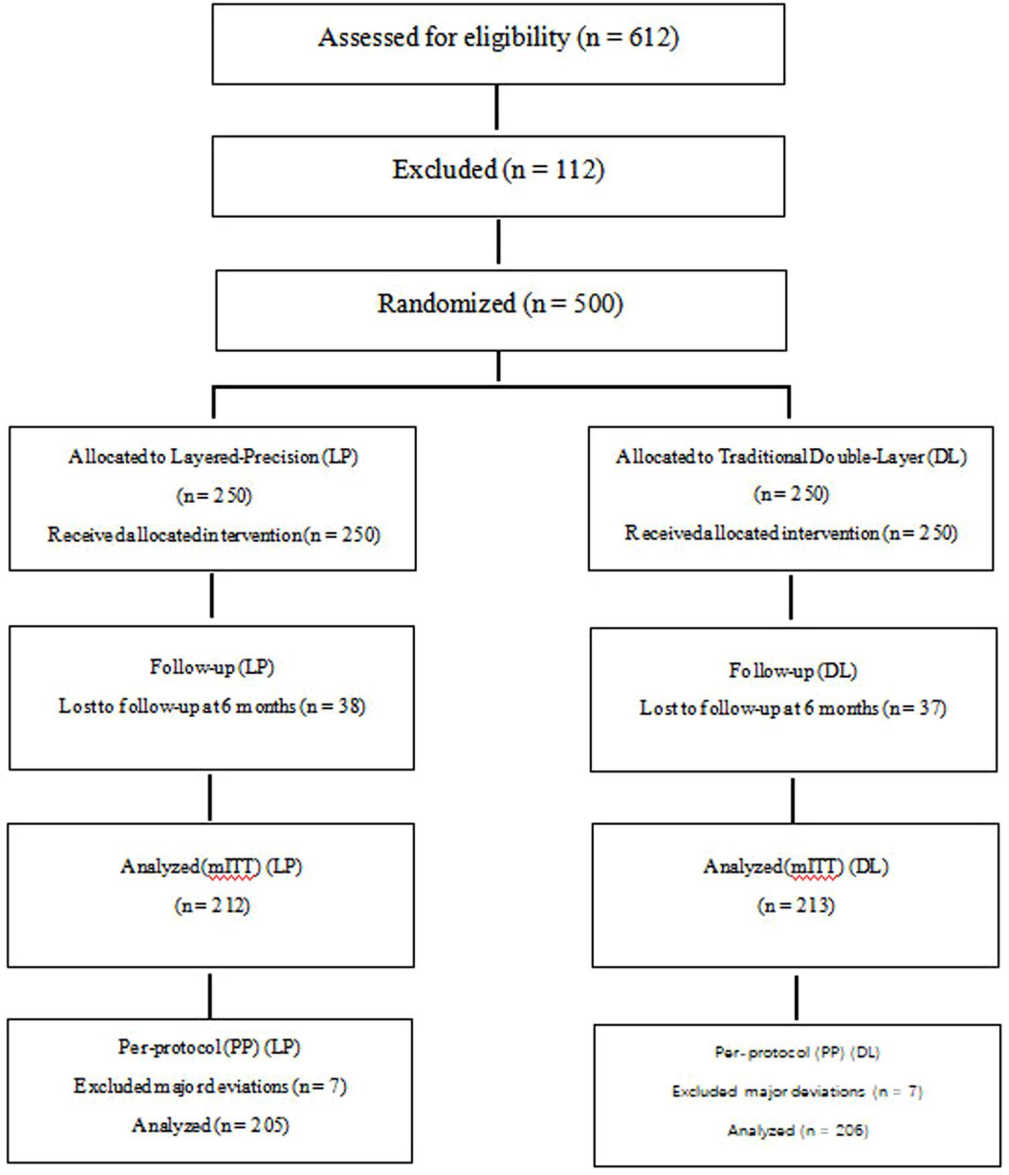
CONSORT flow diagram (editable). mITT includes all randomized participants with primary outcome data; PP excludes prespecified major deviations. Reasons for exclusion and loss to follow-up are documented in the Results section.

Between January and December 2023, 612 individuals were screened; 500 met eligibility criteria and were randomized 1:1 to layered-precision (LP) or traditional DL closure (Figure 1). Of those randomized, all participants (250/250 in LP and 250/250 in DL) received the allocated intervention. By 6 months, primary imaging outcomes were available for 212 of 250 participants in the LP group and 213 of 250 in the DL group, corresponding to approximately 15% missing data per arm. Sensitivity analyses using multiple imputation and per-protocol populations demonstrated consistent treatment effects, suggesting that the impact of missing data on the primary outcome is likely limited. Losses to follow-up and exclusions are summarized in Figure 1, and analysis populations with reasons for exclusion are presented in Supplementary Table S1. The modified intention-to-treat (mITT) population included all randomized participants with primary outcome data (LP: 212, DL: 213). A PP cohort (LP: 205, DL: 206) excluded prespecified major deviations, including non-adherence to technique (*n* = 4 per arm) and imaging outside the prespecified window (*n* = 3 per arm), as detailed in Supplementary Table S1.

### Baseline characteristics

Baseline maternal and obstetric characteristics were similar between groups (Table 1). The mean age was 30.0 ± 4.2 vs. 30.1 ± 4.3 years, and BMI was 26.1 ± 3.9 vs. 26.2 ± 4.0 kg/m^2^ (LP vs. DL, respectively). Indications for cesarean delivery, gestational age (39.0 ± 1.1 vs. 39.1 ± 1.1 weeks), and incision length (10.2 ± 1.0 vs. 10.1 ± 1.0 cm) were balanced between groups; all standardized mean differences were <0.10, with the largest observed for parity (SMD = 0.07). Importantly, comorbid conditions that may influence wound healing, including diabetes mellitus, obesity (BMI ≥30 kg/m^2^), smoking status, and other maternal medical conditions, were also comparable between groups, with all standardized mean differences <0.10 (Table 1). These baseline factors were incorporated into adjusted analyses and considered in the interpretation of healing outcomes.

**Table 1:** Baseline characteristics by randomized group.

Characteristic	LP (*n* = 250)	DL (*n* = 250)	SMD
Age (years)	30.0 ± 4.2	30.1 ± 4.3	0.02
BMI (kg/m^2^)	26.1 ± 3.9	26.2 ± 4.0	0.02
Gestational age at delivery (weeks)	39.0 ± 1.1	39.1 ± 1.1	0.02
Incision length (cm)	10.2 ± 1.0	10.1 ± 1.0	0.04
Parity—nulliparous [*n* (%)]	210 (84.0)	208 (83.2)	
Parity—multiparous [*n* (%)]	40 (16.0)	42 (16.8)	0.07
Indication—labor dystocia [*n* (%)]	85 (34.0)	83 (33.2)	
Indication—fetal intolerance [*n* (%)]	52 (20.8)	51 (20.4)	
Indication—malpresentation [*n* (%)]	38 (15.2)	38 (15.2)	
Indication—elective/other planned [*n* (%)]	75 (30.0)	78 (31.2)	
Diabetes mellitus [*n* (%)]	24 (9.6)	22 (8.8)	0.03
Smoking status [*n* (%)]	17 (6.8)	15 (6.0)	0.03
Other maternal comorbidity [*n* (%)]	29 (11.6)	31 (12.4)	0.02

Values are mean ± SD or *n* (%). SMD, standardized mean difference; all SMDs were <0.10, including for healing-related baseline factors such as diabetes mellitus, smoking status, obesity, and other maternal comorbidities.

### Operative and perioperative outcomes

Operative details and early clinical events are summarized in Table 2. Median EBL was 480 mL [IQR 400–560] in LP vs. 520 mL [IQR 450–600] in DL (difference −40 mL, 95% CI −70 to −10, *P* = 0.009). Operative time was 42.5 ± 8.1 vs. 45.3 ± 8.5 min (difference −2.8 min, 95% CI −4.1 to −1.5, *P* < 0.001). Postoperative fever ≥38°C occurred in 11 of 250 participants in the LP group vs. 18 of 250 in the DL group [risk ratio (RR) 0.61, 95% CI 0.30–1.23; *P* = 0.17], while wound infection occurred in 6 of 250 vs. 10 of 250 (RR 0.60, 95% CI 0.22–1.62; *P* = 0.32). In the LP group, 96% of recorded sutures were ≤30 N, with a mean stitch tension of 24.8 ± 3.6 N.

**Table 2:** Operative and perioperative outcomes.

Outcome	LP (*n* = 250)	DL (*n* = 250)	Effect estimate (95% CI)	*P*-value
Estimated blood loss (mL)	480 [400–560]	520 [450–600]	*Δ* −40 (−70 to −10)	0.009
Operative time (min)	42.5 ± 8.1	45.3 ± 8.5	*Δ* −2.8 (−4.1 to −1.5)	<0.001
Postoperative fever ≥38°C [*n/N* (%)]	11/250 (4.4)	18/250 (7.2)	RR 0.61 (0.30–1.23)	0.17
Wound infection (CDC) [*n/N* (%)]	6/250 (2.4)	10/250 (4.0)	RR 0.60 (0.22–1.62)	0.32
Stitches within cap ≤30 N (LP only) (%)	96	—	—	—
Mean stitch tension, *N* (LP only)	24.8 ± 3.6	—	—	—
Any intraoperative protocol deviation [*n/N* (%)]	7/250 (2.8)	7/250 (2.8)	—	—

Values are median [IQR], mean ± SD, or *n*/*N* (%). *Δ* = difference in central tendency (median for EBL; mean for operative time), LP minus DL. RR = risk ratio for binary outcomes. Stitch-level tension metrics apply only to the LP arm.

### Primary outcomes

**Figure 2:**
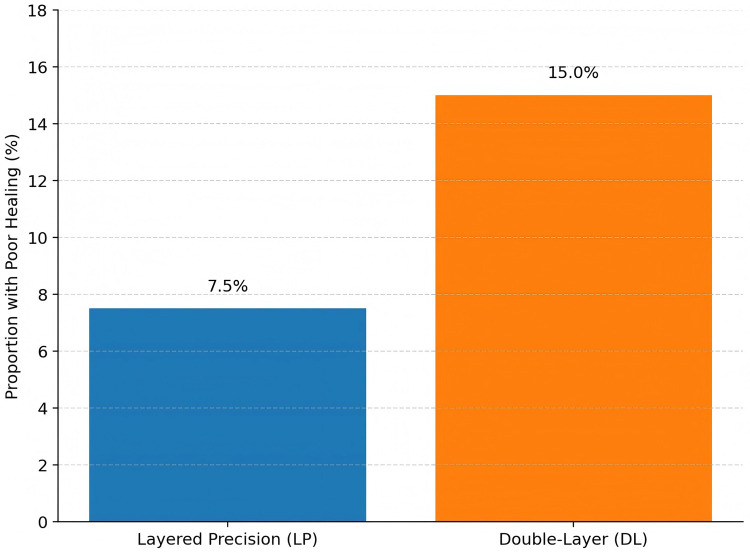
Proportion of participants with poor uterine healing at 6 months, showing a significantly lower rate in the layered precision (LP) group than in the double-layer (DL) group.

At 6 months, poor uterine wound healing (defined as residual myometrial thickness <2.2 mm or the presence of a uterine niche on ultrasound) occurred in 16 of 212 participants (7.5%) in LP vs. 32 of 213 participants (15.0%) in DL (absolute risk difference=7.5 percentage points, 95% CI 1.5–13.4; RR 0.50, 95% CI 0.28–0.89; *P* = 0.015). The adjusted model (including age, BMI, parity, indication, incision length, and baseline comorbidities such as diabetes mellitus, obesity, and smoking) yielded an adjusted risk ratio of 0.50 (95% CI 0.28–0.89; *P* = 0.015), not materially different from the unadjusted estimate. The number needed to treat (NNT) was 13 (95% CI 8–67). Results are summarized in Table 3 and Figure 2.

**Table 3:** Primary outcome at 6 months (poor uterine wound healing).

Outcome	LP (*n* = 212)	DL (*n* = 213)	Absolute risk difference, pp (95% CI)	RR (95% CI)	aRR (95% CI)	*P*-value
Poor healing [*n*/*N* (%)]	16/212 (7.5)	32/213 (15.0)	7.5 (1.5–13.4)	0.50 (0.28–0.89)	0.50 (0.28–0.89)	0.015
NNT (95% CI)	—	—	13 (8–67)	—	—	—

Poor uterine wound healing was defined as residual myometrial thickness <2.2 mm or the presence of a uterine niche on standardized ultrasound. pp, percentage point; RR, risk ratio; aRR, adjusted risk ratio (adjusted for age, BMI, parity, indication, incision length).

### Imaging outcomes

**Figure 3:**
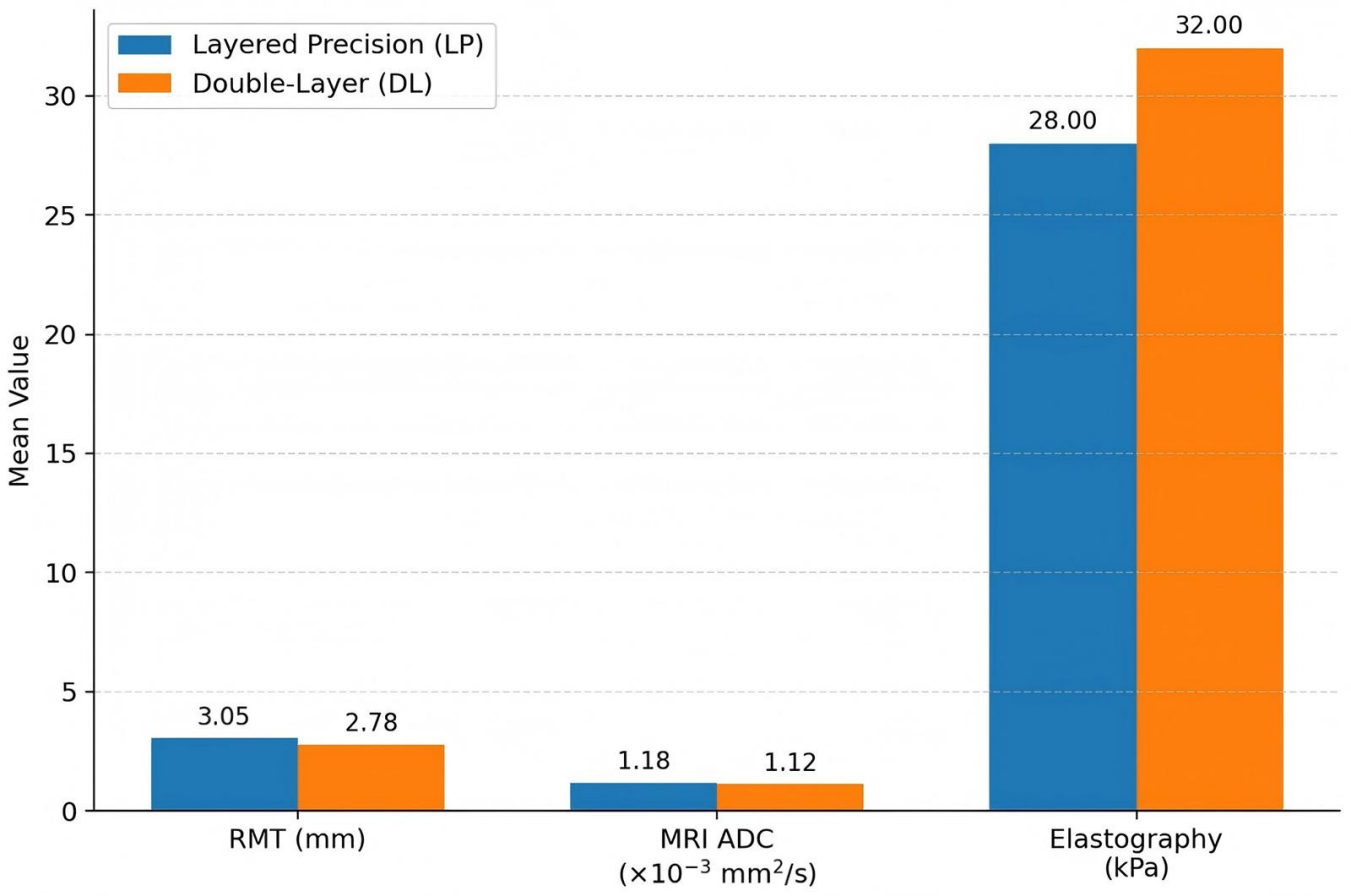
Comparison of imaging metrics at 6 months, showing greater residual myometrial thickness and higher MRI ADC but lower elastography stiffness in the layered precision (LP) group versus the double-layer (DL) group.

**Figure 4:**
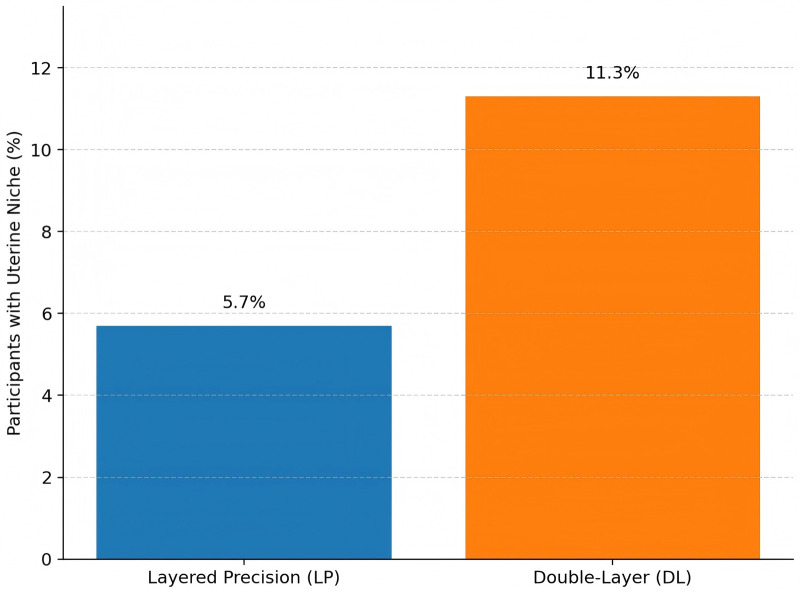
Niche prevalence at 6 months, showing fewer participants with a uterine niche in the layered precision (LP) group (5.7%) than in the double-Layer (DL) group (11.3%).

Continuous imaging metrics are presented in Table 4 and Figures 3, 4. Mean RMT was 3.05 ± 0.62 mm vs. 2.78 ± 0.65 mm (adjusted mean difference 0.27 mm, 95% CI 0.12–0.42; *P* < 0.001). Niche prevalence was 12 of 212 vs. 24 of 213 (RR 0.50, 95% CI 0.26–0.98; *P* = 0.043). In exploratory adjunct imaging analyses, mean ADC values in the MRI subset (LP: 64; DL: 64) were 1.18 ± 0.12 vs. 1.12 ± 0.13 × 10⁻^3^ mm^2^/s (difference 0.06, 95% CI 0.02–0.10; *P* = 0.004). Similarly, in the exploratory elastography subset (LP: 32; DL: 32), median stiffness was 28 kPa [IQR 24–33] vs. 32 kPa [IQR 27–37] (difference −4 kPa, 95% CI −6 to −2; *P* = 0.001). These findings are explicitly exploratory and hypothesis-generating and should not be considered confirmatory evidence.

**Table 4:** Imaging metrics at 6 months.

Metric	LP	DL	*Δ* or Ratio (95% CI)	*P*-value
RMT, mm, mean ± SD	3.05 ± 0.62	2.78 ± 0.65	*Δ* 0.27 (0.12–0.42)	<0.001
Niche present [*n/N* (%)]	12/212 (5.7)	24/213 (11.3)	RR 0.50 (0.26–0.98)	0.043
MRI ADC ×10⁻^3^ mm^2^/s, mean ± SD*	1.18 ± 0.12 (*n* = 64)	1.12 ± 0.13 (*n* = 64)	*Δ* 0.06 (0.02–0.10)	0.004
Elastography, kPa, median [IQR]^†^	28 [24–33] (*n* = 32)	32 [27–37] (*n* = 32)	*Δ* −4 (−6 to −2)	0.001

*MRI subset only. ^†^Elastography optional subset. *Δ* = difference (LP−DL); RR = risk ratio.

### Patient-reported outcomes

Completion rates for PROs were 95% at discharge, 90% at 6 weeks, and 85% at 6 months (Table 5). Pain scores (VAS 0–10) were 3.9 vs. 4.5 at discharge (adjusted difference −0.6, 95% CI −0.9 to −0.3; *P* < 0.001), 2.1 vs. 2.5 at 6 weeks (−0.4, 95% CI −0.6 to −0.2; *P* < 0.001), and 0.8 vs. 1.0 at 6 months (−0.2, 95% CI −0.4 to 0.0; *P* = 0.052) for LP vs. DL, respectively. At 6 months, sexual quality-of-life scores were 78.6 ± 10.8 in the LP group and 76.9 ± 11.2 in the DL group (adjusted difference 1.7, 95% CI −0.3 to 3.7; *P* = 0.094), while menstrual pattern change scores were 2.3 ± 1.1 vs. 2.6 ± 1.2 (adjusted difference −0.3, 95% CI −0.6 to 0.0; *P* = 0.071). These PRO differences are reported quantitatively but did not reach statistical significance.

**Table 5:** Patient-reported outcomes.

Outcome	LP	DL	Adjusted difference (95% CI)	*P*-value
PRO completion at discharge [*n*/*N* (%)]	238/250 (95.2)	237/250 (94.8)	—	—
PRO completion at 6 weeks [*n*/*N* (%)]	226/250 (90.4)	224/250 (89.6)	—	—
PRO completion at 6 months [*n*/*N* (%)]	212/250 (84.8)	213/250 (85.2)	—	—
Pain VAS (0–10) at discharge	3.9	4.5	*Δ* −0.6 (−0.9 to −0.3)	<0.001
Pain VAS (0–10) at 6 weeks	2.1	2.5	*Δ* −0.4 (−0.6 to −0.2)	<0.001
Pain VAS (0–10) at 6 months	0.8	1.0	*Δ* −0.2 (−0.4 to 0.0)	0.052
Sexual QoL score at 6 months	78.6 ± 10.8	76.9 ± 11.2	*Δ* 1.7 (−0.3 to 3.7)	0.094
Menstrual pattern change score at 6 months	2.3 ± 1.1	2.6 ± 1.2	*Δ* −0.3 (−0.6 to 0.0)	0.071

PRO completion rates are shown by randomized group. Pain scores are means on a 0–10 VAS. Sexual QoL and menstrual pattern change scores are presented as mean ± SD. Adjusted differences control for age, BMI, parity, indication, and incision length.

### Subsequent pregnancy outcomes

During follow-up, 54 of 250 participants (21.6%) in LP and 52 of 250 participants (20.6%) in DL conceived; obstetric outcomes are summarized in Table 6. Placenta accreta spectrum occurred in 2 of 54 participants (3.7%) vs. 5 of 52 participants (9.6%) (RR 0.39, 95% CI 0.08–1.90), and uterine rupture/dehiscence occurred in 1 of 54 participants (1.9%) vs. 3 of 52 participants (5.8%) (RR 0.32, 95% CI 0.03–3.00). Vaginal birth after cesarean (VBAC) occurred in 18 of 54 participants (33.3%) in the LP group and 15 of 52 participants (28.8%) in the DL group (RR 1.16, 95% CI 0.65–2.06; *P* = 0.62), whereas repeat cesarean delivery occurred in 36 of 54 participants (66.7%) and 37 of 52 participants (71.2%), respectively. Neonatal composite adverse outcomes occurred in 6 of 54 participants (11.1%) in the LP group and 8 of 52 participants (15.4%) in the DL group (RR 0.72, 95% CI 0.27–1.89; *P* = 0.50). No statistically significant between-group differences were observed for pregnancy or neonatal outcomes.

**Table 6:** Subsequent pregnancy outcomes (during follow-up).

Outcome	LP	DL	Effect estimate (95% CI)	*P*-value
Any subsequent pregnancy [*n*/*N* (%)]	54/250 (21.6)	52/250 (20.8)	RR 1.04 (0.74–1.46)	0.83
Placenta accreta spectrum [*n*/*N* (%)]	2/54 (3.7)	5/52 (9.6)	RR 0.39 (0.08–1.90)	0.25
Uterine rupture/dehiscence [*n*/*N* (%)]	1/54 (1.9)	3/52 (5.8)	RR 0.32 (0.03–2.99)	0.32
Mode of birth (VBAC) [*n*/*N* (%)]	18/54 (33.3)	15/52 (28.8)	RR 1.16 (0.65–2.06)	0.62
Repeat cesarean delivery [*n*/*N* (%)]	36/54 (66.7)	37/52 (71.2)	RR 0.94 (0.72–1.22)	0.62
Neonatal composite adverse outcome [*n*/*N* (%)]	6/54 (11.1)	8/52 (15.4)	RR 0.72 (0.27–1.89)	0.50

Effect estimates are risk ratios (RRs) with 95% confidence intervals. Denominators for placenta accreta spectrum, uterine rupture/dehiscence, mode of birth, and neonatal outcomes are the number of participants with a subsequent pregnancy in each group.

### Safety

Adverse events are summarized in Table 7. Any AE occurred in 42 of 250 participants in the LP group vs. 49 of 250 participants in the DL group (RR 0.86, 95% CI 0.59–1.24), while serious AEs occurred in 4 of 250 vs. 6 of 250 (RR 0.67, 95% CI 0.19–2.33). The most frequently observed adverse events included postoperative fever, wound infection, secondary postpartum hemorrhage, hospital readmission, and reoperation-related events. Serious adverse events were predefined as major hemorrhage requiring transfusion or procedural intervention, severe infection requiring intravenous antibiotics or readmission, unplanned reoperation, or other clinically significant complications requiring prolonged hospitalization. No clear excess of any specific serious adverse event category was observed in the LP group compared with the DL group. The data and safety monitoring board (DSMB) did not recommend protocol modifications, and no stopping boundaries for harm or futility were crossed.

**Table 7:** Adverse events (safety population).

AE category	LP [*n*/*N* (%)]	DL [*n*/*N* (%)]	RR (95% CI)	*P*-value
Any AE	42/250 (16.8)	49/250 (19.6)	0.86 (0.59–1.24)	—
Postoperative fever	11/250 (4.4)	18/250 (7.2)	0.61 (0.30–1.23)	0.17
Wound infection	6/250 (2.4)	10/250 (4.0)	0.60 (0.22–1.62)	0.32
Secondary postpartum hemorrhage	9/250 (3.6)	8/250 (3.2)	1.13 (0.44–2.89)	0.80
Hospital readmission	8/250 (3.2)	9/250 (3.6)	0.89 (0.35–2.28)	0.81
Reoperation-related events	4/250 (1.6)	6/250 (2.4)	0.67 (0.19–2.33)	0.52
Serious AE	4/250 (1.6)	6/250 (2.4)	0.67 (0.19–2.33)	—
Major hemorrhage requiring transfusion or procedural intervention	2/250 (0.8)	3/250 (1.2)	0.67 (0.11–3.93)	0.66
Severe infection requiring IV antibiotics or readmission	1/250 (0.4)	2/250 (0.8)	0.50 (0.05–5.46)	0.57
Unplanned reoperation for postoperative complication	1/250 (0.4)	1/250 (0.4)	1.00 (0.06–15.85)	1.00

AE, adverse event; RR, risk ratio. Serious adverse events were predefined as major hemorrhage requiring transfusion or procedural intervention, severe infection requiring intravenous antibiotic therapy or readmission, unplanned reoperation, or other clinically significant complications requiring prolonged hospitalization.

### Subgroup and sensitivity analyses

**Figure 5:**
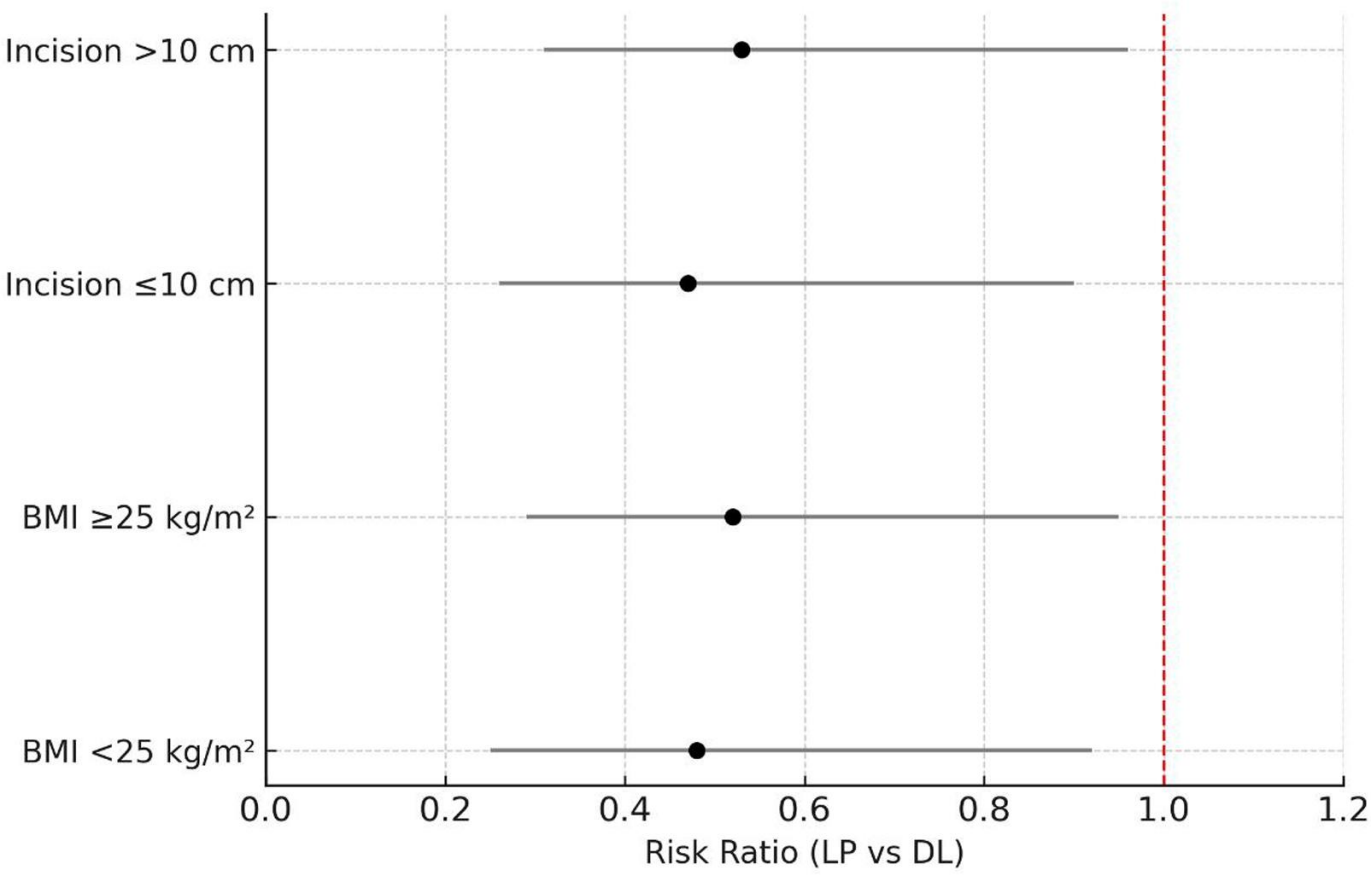
Subgroup analyses for poor uterine healing at 6 months. Risk ratios (RRs) and 95% confidence intervals are shown for prespecified subgroups by body mass index (BMI) and incision length. All subgroup estimates favored the layered precision (LP) technique, with no significant interaction between subgroups (*P* for interaction = 0.42 and 0.58).

Prespecified subgroup analyses (BMI <25 vs. ≥25 kg/m^2^; incision length ≤10 vs. >10 cm) showed no significant interaction with treatment effect (*P* interaction = 0.42 and 0.58, respectively), with point estimates favoring the layered-precision (LP) arm across strata (Figure 5). Sensitivity analyses using the PP cohort and multiple-imputation datasets produced results consistent with the primary analysis (Supplementary Tables S1–S3).

## Discussion

In this randomized trial, LP uterine closure with quantified tension control halved the 6-month rate of poor healing vs. DL closure (7.5% vs. 15.0%), increased RMT, lowered niche prevalence, and showed concordant gains on MRI ADC and shear-wave elastography; perioperative outcomes and safety were similar. These effects were consistent across prespecified subgroups and sensitivity analyses. Importantly, these findings demonstrate improvement in imaging-based markers of uterine scar healing, which serve as surrogate endpoints rather than direct clinical endpoints. Although MRI ADC and shear-wave elastography findings were directionally concordant with the ultrasound results, they were obtained in smaller feasibility-based subsets and should be regarded as exploratory adjunct evidence rather than confirmatory.

Post-2020 evidence increasingly questions whether “how many layers” matters as much as “how layers are handled.” The 3-year follow-up of the 2Close randomized trial reported no superiority of DL over single-layer for reproductive outcomes after a first cesarean, arguing against a default preference for DL on long-term endpoints (5). Recent meta-analyses have also shown that, at early imaging timepoints, single-layer closure can yield comparable or better scar morphology relative to DL, reinforcing that ischemic compression and tissue handling, rather than nominal layer count, likely drive remodeling quality (17). This evolving perspective has also been highlighted in the recent *AJOG* review by Bujold and Romero, which synthesized the biological mechanisms of uterine healing and emphasized the importance of surgical biomechanics, including tissue apposition, perfusion preservation, and avoidance of excessive compression during closure. While that review provided a conceptual and mechanistic framework, it underscored the lack of prospective randomized studies directly evaluating surgical techniques that operationalize these biomechanical principles. Our findings extend this literature by standardizing bite length across planes and capping suture tension at ≤30 N using a calibrated handheld gauge (selected based on preliminary feasibility testing and tissue model simulations to balance apposition and avoid excessive compression), which translates these biological and biomechanical principles into measurable improvements in uterine scar healing, without adversely affecting perioperative outcomes.

### Clinical meaning of the imaging endpoints

We defined poor healing using contemporary niche terminology—an indentation ≥2 mm on transvaginal/3D ultrasound—now reflected in recent consensus guidance and reviews; this approach enhances comparability across studies and links imaging findings to counseling regarding symptoms and future pregnancy outcomes (18–20). The thicker RMT and lower niche prevalence observed in our LP group align with reproducibility advances in 3D cesarean-scar sonography, where standardized acquisition has improved the reliability of RMT and volume metrics (21). The higher ADC values we observed in the LP group are biologically plausible and consistent with gynecologic MRI literature that supports DWI/ADC as a quantitative marker of tissue microstructure, provided that harmonized protocols are used (22, 23). Elastography data remain emergent; however, our lower stiffness findings in the LP subset is consistent with recent reports showing the feasibility and potential discriminative value of shear-wave techniques in cesarean scar pathology (12). However, because MRI and elastography were performed in limited subsets, these findings should be interpreted cautiously as exploratory adjunct evidence rather than definitive confirmation of the primary ultrasound-based outcome. Nevertheless, these imaging parameters serve as surrogate indicators of uterine scar quality, and their relationship to subsequent reproductive outcomes, such as fertility, uterine rupture, or placenta accreta spectrum, remains an area of ongoing investigation.

### Operative and perioperative course

LP modestly reduced estimated blood loss and operative time without increasing postoperative fever or wound infection. These findings are consistent with technique-focused literature indicating that minimizing tissue compression and standardizing passes can shorten operative steps without consistent tradeoffs in short-term morbidity; our study adds randomized evidence with quantified tension control (17).

### Patient-reported outcomes

Pain scores favored LP at discharge and 6 weeks, converging by 6 months—a recovery pattern consistent with literature linking nich formation and reduced RMT to abnormal bleeding and pelvic pain. Sexual quality-of-life and menstrual-pattern change scores are reported quantitatively, showing directionally favorable but non-significant differences for LP. These outcomes are secondary, and the trial was not powered to detect clinically meaningful differences in symptom-based endpoints (18, 19).

### Subsequent pregnancy

Early trends for placenta accreta spectrum and uterine rupture/dehiscence favored the LP group but were rare and imprecise; this mirrors recent syntheses that emphasize that definitive reproductive endpoints require larger sample sizes and longer follow-up. The present trial was not powered to evaluate obstetric outcomes such as uterine rupture, placenta accreta spectrum, or fertility-related endpoints, and the observed differences should therefore be interpreted cautiously as exploratory. The 3-year data of the 2Close trial similarly found no advantage of DL closure for live-birth outcomes, supporting our inference that improving scar biology may be more impactful than adding a layer *per se*, while reaffirming the need for multicenter confirmation (5, 17, 24).

### Mechanistic considerations

The layer-specific bite length and ≤30 N tension cap of the LP protocol target two key mechanistic levers—apposition and perfusion—that plausibly reduce ischemic damage and optimize remodeling. The ≤30 N threshold was chosen based on preliminary feasibility testing and tissue model simulations; the handheld gauge was calibrated, and surgeons were trained to ensure reproducible measurements. While feasible in this trial, generalizability to routine clinical practice remains to be established. The concordance of ultrasound findings with exploratory MRI and elastography results is mechanistically reassuring; however, these adjunct imaging data should not be interpreted as confirmatory, as the study was not powered for these subset analyses. These findings are consistent with mechanistic principles summarized in the *AJOG* review, which emphasized that restoration of myometrial anatomy and preservation of tissue perfusion are central determinants of uterine scar healing (6). Our results provide prospective clinical evidence supporting the translational application of these principles in surgical technique design.

### Strengths

Strengths of this study include a prospectively registered RCT, standardized surgeon training, intraoperative tension quantification, blinded imaging adjudication, guideline-concordant definitions for niche and RMT, and complementary MRI (DWI/ADC) with optional elastography.

### Limitations

This single-center design may limit generalizability across surgical settings and devices. Although the reproducibility of 3D ultrasound has improved, residual measurement variability remains inevitable in ultrasound-based assessment of the niche and RMT. MRI and elastography were performed in feasibility-based subsets, and the study was not powered to establish these modalities as confirmatory endpoints; accordingly, these findings should be interpreted as exploratory. Surgeons were necessarily unblinded, so unmeasured nuances in surgical handling could remain. In addition, because the layered-precision arm incorporated a more protocolized operative approach, including structured training, procedural checklists, and intraoperative tension monitoring, part of the observed effect may reflect procedural standardization rather than the closure technique alone. Although multiple surgeons contributed to both groups and exploratory analyses considered operator-level variability, performance bias related to protocolized surgery cannot be excluded. The tension threshold used was based on feasibility testing and has not been formally validated; its reproducibility and applicability in routine clinical practice remain to be determined. Approximately 15% of participants were lost to follow-up for the primary 6-month imaging outcome. Although this attrition was anticipated in the sample size calculation, it introduces the potential for attrition bias if losses were systematically related to group allocation or underlying wound-healing characteristics. We attempted to mitigate this risk by using multiple imputation for missing primary outcome data and conducting sensitivity analyses using both per-protocol and complete-case datasets. The consistency of findings across these analyses supports the robustness of the primary results, although caution is warranted when interpreting absolute risk estimates. Finally, the trial was not powered to evaluate long-term obstetric outcomes, and rare obstetric outcomes [e.g., rupture, placenta accreta spectrum (PAS)] require longer follow-up and multicenter samples for precise estimation (22–24). As a single-center study, the generalizability of these findings to other institutions or global cesarean practice is limited. Future multicenter trials should incorporate surgeon-level clustering analyses and evaluate learning curves to better isolate the independent effect of closure technique from procedural standardization.

### Clinical implications

Together with post-2020 RCT follow-up and meta-analysis evidence, our findings suggest that surgical technique during uterine closure, including tissue handling and suture tension control, may influence quantitative markers of uterine scar healing. The layered precision approach evaluated in this trial provides a standardized method to operationalize these principles and was associated with improved imaging-based measures of scar morphology without increasing perioperative complications. These findings should be interpreted cautiously, as they reflect surrogate indicators of scar quality rather than evidence of improved reproductive or obstetric outcomes. While structured training with procedural checklists and tension feedback may facilitate reproducible implementation of such techniques, whether these morphological improvements translate into clinically meaningful benefits, such as reduced symptoms, improved fertility, or lower risk of complications in subsequent pregnancies, requires confirmation in larger studies with longer follow-up. Given converging guidance on niche definitions and the growing role of quantitative ultrasound and MRI, standardized imaging assessment (RMT and 3D morphology, with optional ADC) may support consistent follow-up and research evaluation. Elastography remains investigational until validated thresholds are established (17, 22, 23).

### Future directions

Multicenter pragmatic RCTs are needed to test scalability across varying levels of surgeon experience and different devices, to formally link imaging gains to bleeding, pain, and fertility, and to predefine clinically meaningful thresholds for RMT, ADC, and scar stiffness. Future studies should also evaluate the reproducibility and clinical utility of suture tension monitoring across different surgical environments and devices, while accounting for surgeon-level clustering and learning curve effects to distinguish the independent impact of the technique from procedural standardization. Centralized imaging protocols/reading will help further reduce variability (17, 18, 22, 23).

## Conclusions

In this single-center study, LP closure with quantified tension control improved quantitative imaging markers of uterine scar healing, including RMT, niche prevalence, and complementary MRI and elastography indices, without increasing perioperative complications. These findings provide evidence of improved scar morphology but do not establish benefits in definitive patient-centered reproductive outcomes. Multicenter trials with longer follow-up are needed to determine clinical significance.

## Data Availability

The raw data supporting the conclusions of this article will be made available by the authors, without undue reservation.
