# Supplementary material for: Layered precision suturing vs. traditional double-layer closure at cesarean: a randomized trial of uterine scar healing and maternal outcomes
Source: Front Surg. 2026 May 4;13:1749613. doi: 10.3389/fsurg.2026.1749613 (PMC13180858; doi:10.3389/fsurg.2026.1749613)
Supplement: Supplementary file 1 [file Table1.docx]

**Supplementary Tables**

**Table S1. Analysis populations and exclusions**

| **Population** | **LP n** | **DL n** | **Total n** | **Notes / Reasons for exclusion** |
| --- | --- | --- | --- | --- |
| Randomized | 250 | 250 | 500 | — |
| Safety population (received intervention) | 250 | 250 | 500 | All received allocated technique |
| mITT (primary outcome available) | 212 | 213 | 425 | Primary ultrasound at 6 months within window |
| PP (major deviations excluded) | 205 | 206 | 411 | Excluded: non-adherence to technique (n=4 per arm), imaging outside window (n=3 per arm) |

Abbreviations: mITT = modified intention-to-treat; PP = per protocol. Major deviations were prespecified in the protocol.

**Table S2. Prespecified subgroup analyses for the primary outcome**

| **Subgroup** | **LP n/N (%)** | **DL n/N (%)** | **RR (95% CI)** | **P for interaction** | **Direction** |
| --- | --- | --- | --- | --- | --- |
| BMI <25 kg/m² | 5/90 (5.6%) | 12/92 (13.0%) | 0.43 (0.16–1.16) | 0.42 | Favors LP |
| BMI ≥25 kg/m² | 11/122 (9.0%) | 20/121 (16.5%) | 0.55 (0.27–1.09) | — | Favors LP |
| Incision length ≤10 cm | 7/110 (6.4%) | 15/112 (13.4%) | 0.48 (0.20–1.12) | 0.58 | Favors LP |
| Incision length >10 cm | 9/102 (8.8%) | 17/101 (16.8%) | 0.52 (0.25–1.12) | — | Favors LP |

Interaction P values are shown once per subgroup family (BMI and incision length). All estimates use the mITT population.

**Table S3. Sensitivity analyses for the primary outcome**

| **Analysis set** | **LP n/N (%)** | **DL n/N (%)** | **RR (95% CI)** | **Adjusted RR (95% CI)** | **Conclusion** |
| --- | --- | --- | --- | --- | --- |
| mITT (primary) | 16/212 (7.5%) | 32/213 (15.0%) | 0.50 (0.28–0.89) | 0.50 (0.28–0.89) | Consistent; favors LP |
| Per protocol (PP) | 14/205 (6.8%) | 29/206 (14.1%) | 0.49 (0.26–0.89) | 0.49 (0.27–0.88) | Consistent; favors LP |
| Multiple imputation (primary outcome) | 16/212 (7.5%) | 32/213 (15.0%) | 0.50 (0.28–0.89) | 0.50 (0.28–0.89) | Consistent; favors LP |
